# Influenza Virus Hemagglutinin Stalk-Specific Antibodies in Human Serum are a Surrogate Marker for *In Vivo* Protection in a Serum Transfer Mouse Challenge Model

**DOI:** 10.1128/mBio.01463-17

**Published:** 2017-09-19

**Authors:** Henning Jacobsen, Madhusudan Rajendran, Angela Choi, Haakon Sjursen, Karl A. Brokstad, Rebecca J. Cox, Peter Palese, Florian Krammer, Raffael Nachbagauer

**Affiliations:** a Department of Microbiology, Icahn School of Medicine at Mount Sinai, New York, New York, USA; b Graduate School of Biological Sciences, Icahn School of Medicine at Mount Sinai, New York, New York, USA; c Influenza Centre, Department of Clinical Science, University of Bergen, Bergen, Norway; d Section for Infectious Diseases, Medical Department, Haukeland University Hospital, Bergen, Norway; e Department of Clinical Science, Broeglemann Research Laboratory, University of Bergen, Bergen, Norway; f KG Jebsen Centre for Influenza Vaccine Research, Department of Clinical Science, University of Bergen, Bergen, Norway; g Department of Research and Development, Haukeland University Hospital, Bergen, Norway; GSK Vaccines

**Keywords:** ADCC, ELISA, correlate of protection, hemagglutinin, hemagglutinin stalk, influenza, influenza vaccines, surrogate marker, universal influenza virus vaccine

## Abstract

The immunogenicity of current influenza virus vaccines is assessed by measuring an increase of influenza virus-specific antibodies in a hemagglutination inhibition assay. This method exclusively measures antibodies against the hemagglutinin head domain. While this domain is immunodominant, it has been shown that hemagglutination inhibition titers do not always accurately predict protection from disease. In addition, several novel influenza virus vaccines that are currently under development do not target the hemagglutinin head domain, but rather more conserved sites, including the hemagglutinin stalk. Importantly, antibodies against the hemagglutinin stalk do not show activity in hemagglutination inhibition assays and will require different methods for quantification. In this study, we tested human serum samples from a seasonal influenza virus vaccination trial and an avian H5N1 virus vaccination trial for antibody activities in multiple types of assays, including binding assays and also functional assays. We then performed serum transfer experiments in mice which then received an H1N1 virus challenge to assess the *in vivo* protective effects of the antibodies. We found that hemagglutinin-specific antibody levels measured in an enzyme-linked immunosorbent assay (ELISA) correlated well with protection from weight loss in mice. In addition, we found that weight loss was also inversely correlated with the level of serum antibody-dependent cellular cytotoxicity (ADCC) as measured in a reporter assay. These findings indicate that protection is in part conferred by Fc-dependent mechanisms. In conclusion, ELISAs can be used to measure hemagglutinin-specific antibody levels that could serve as a surrogate marker of protection for universal influenza virus vaccines.

## IMPORTANCE

Influenza viruses are a serious concern for public health and cause a large number of deaths worldwide every year. Current influenza virus vaccines can confer protection from disease, but they often show low efficacy due to the ever-changing nature of the viruses. Novel vaccination approaches target conserved epitopes of the virus, including the hemagglutinin stalk domain, to elicit universally protective antibodies that also bind to mutated viruses or new subtypes of viruses. Importantly, the hemagglutination inhibition assay—the only assay that has been accepted as a correlate of protection by regulatory authorities—cannot measure antibodies against the hemagglutinin stalk domain. Therefore, novel correlates of protection and assays to measure vaccine immunogenicity need to be developed. In this study, we correlated the results from multiple assays with protection in mice after transfer of human serum and a lethal virus challenge to investigate potential novel serological surrogate markers for protection.

## INTRODUCTION

Influenza viruses can cause severe human respiratory disease and are a significant burden to public health (1, 2). Seasonal influenza virus vaccines are currently the most effective form of prophylaxis against disease (3). These vaccines elicit strain-specific antibody responses that predominantly target the immunodominant head domain of influenza virus hemagglutinin (HA) (4). The titers of antibodies against this domain can be measured in a hemagglutination inhibition assay (HAI). HAI titers of 1:40 have been shown to correlate with a 50% reduction in infection and are commonly used as an indicator for successful seroconversion in influenza virus vaccination trials (5–7). This protective titer was originally established in healthy adults with specific virus strains and does not necessarily apply to all age groups or to all influenza viruses. Importantly, in case of suboptimal cellular immunity, particularly in children and elderly individuals, substantially higher HAI titers might be required for similar levels of protection (8–10). Recent advances in influenza virus research have resulted in novel candidate vaccines that are aimed at inducing broader protection from influenza virus infection. Notably, some of these approaches focus on shifting the antibody response away from the HA head domain and toward the conserved HA stalk region. Antibodies against the HA stalk region cannot be detected in the traditional HAI assay, and so it is necessary to develop novel assays to quantify the protective effects conferred by these antibodies (4, 11–14).

HA stalk-specific antibodies can be detected in enzyme-linked immunosorbent assays (ELISAs). However, ELISAs only measure antigen binding and cannot predict whether these antibodies are functional. HA stalk-specific antibodies can mediate their function through various mechanisms. These functions can be measured in neutralization assays and reporter assays that measure Fc receptor activation (15, 16). Furthermore, HA stalk-specific antibodies can also mediate neuraminidase inhibitory activity (17, 18). Ideally, these assays could be used to predict antibody-mediated protection, similar to measurements from HAI assays.

To see if *in vitro* binding and functional assays correlate with protection *in vivo*, we tested human serum samples from two influenza virus vaccination trials in multiple assays and correlated the results with weight loss in a serum transfer mouse challenge model.

## RESULTS

### H5N1 vaccination induces high levels of H1-reactive antibodies that are not active in hemagglutination inhibition.

Serum samples from an H5N1 vaccination trial before and after one or two vaccinations (with or without adjuvant) were used for this study. Importantly, the vaccination trial started in early 2009 in Norway, before the 2009 H1N1 pandemic virus (H1N1pdm09) had been introduced into the local population (19, 20). The study participants were therefore likely not previously exposed to the virus proteins used in our assays. A cohort of Norwegian health care workers who received a trivalent seasonal influenza virus vaccine (TIV) in 2014 was included as a positive control. This group likely had prior exposure to H1N1pdm09 virus, through adjuvanted pandemic vaccination in 2009, and the strain was included in the seasonal vaccine.

**FIG 1:**
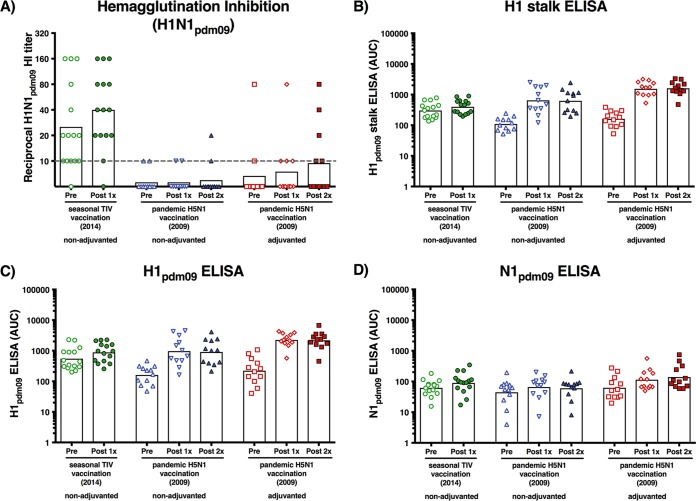
Hemagglutination inhibition assay and ELISA results. Serum samples from individuals pre- and postvaccination were measured individually. Serum samples are shown as individual symbols. Bars represent geometric mean titers. (A) Hemagglutination inhibition was measured against H1N1pdm09 virus. The gray dashed line indicates the limit of detection. (B) Hemagglutinin stalk-specific antibodies were measured by ELISA using a recombinantly produced cH6/1 hemagglutinin. The construct contains an exotic hemagglutinin head domain to which humans are generally not exposed and the H1N1pdm09 hemagglutinin stalk domain. (C) Antibodies against H1N1pdm09 hemagglutinin were measured against recombinantly produced protein. (D) Antibodies against H1N1pdm09 neuraminidase were measured against recombinantly produced protein.

We confirmed that the TIV group had preexisting antibodies by using an HAI assay that measured antibodies against the HA head domain, which are generally highly subtype specific. After vaccination, the antibody titers increased to a geometric mean titer (GMT) of 1:40 (95% confidence interval [CI], 22.4 to 71.5). In contrast, individuals in both the adjuvanted and nonadjuvanted H5N1 vaccination groups had very low titers against the H1 head domain before vaccination (GMT of 1:6.7 and 1:5.6, respectively), and titers did not increase substantially after H5N1 vaccination (GMT of 1:9.4 and 1:5.9, respectively). This result was expected, since the HA head domains of H1 and H5 are antigenically distinct. Three individuals in the adjuvanted H5N1 vaccination group exhibited increased titers against H1 after vaccination, which could indicate some induction of head cross-reactive antibodies (Fig. 1A).

Next, an ELISA with a chimeric HA construct that contained an exotic head domain of H6 and the stalk domain of H1 was performed. Since humans are generally naive to the H6 head domain, this assay allows specific measurement of antibodies against the H1 stalk domain (Fig. 1B). H1 stalk-specific antibodies were strongly induced after two vaccinations in both the adjuvanted (9.9-fold geometric mean increase [GMI]; 95% CI, 6.1 to 16.0) and nonadjuvanted (5.7-fold GMI; 95% CI, 3.0 to 10.7) H5N1 groups. This strong antibody response in H5N1 vaccinees was most likely caused by a recall of memory B cells that target conserved epitopes in the HA stalk domain. This preexisting pool of memory B cells with specificities to the HA stalk domain was likely generated by prior exposures to pre-pandemic seasonal influenza viruses. The previous exposures were further indicated by prevaccination antibody levels against the H1 stalk domain. In the absence of preexisting memory cells against H5 head epitopes, these cells overcome the often-observed dominance of HA head-specific B cells (21, 22). The increase of H1 stalk-reactive antibodies in the TIV vaccination group was low (1.3-fold GMI; 95% CI, 1.1 to 1.5), which is consistent with an antibody response that preferentially targets the HA head domain (23).

To measure the combined HA head and stalk responses, an ELISA against full-length H1pdm09 was performed. The antibody levels measured in this assay were mostly consistent with the results from the stalk-specific assay, with slightly greater induction (1.6-fold GMI; 95% CI, 1.3 to 2.0) in the TIV group, which could account for the induction of head-specific antibodies (Fig. 1C). A correlation analysis of HI titers and H1 ELISA antibody levels revealed that a high HI titer also resulted in high levels of antibodies measured by ELISA in the seasonal vaccination cohort. In the H5N1 vaccination cohorts (with low HI titers), no correlation between HI and ELISA titers was observed (see Fig. S1A to C in the supplemental material). When ELISA results for full-length HA were correlated with antibody levels specific to the HA stalk, a very good correlation was observed in the H5N1 vaccination cohorts, indicating that the H1 antibody response was almost exclusively mediated against the HA stalk (Fig. S1E and F). A slightly lower correlation was observed for the seasonal vaccination cohort, which is consistent with an antibody repertoire against both the HA head and stalk domains (Fig. S1D).

10.1128/mBio.01463-17.1 FIG S1  Correlation of HI and ELISA results. (A to C) To test if individuals with high HI titers also showed high titers against H1 (measured by ELISA), the results were correlated for the three vaccination cohorts separately. (D to F) To find the proportion of antibodies of HA stalk-specific antibodies measured in the full-length H1 ELISA, the results of the full-length ELISAs were correlated with the results measured with the cH6/1 construct. Download FIG S1, TIF file, 0.6 MB. Copyright © 2017 Jacobsen et al. 2017 Jacobsen et al. This content is distributed under the terms of the Creative Commons Attribution 4.0 International license.

An additional ELISA that measured antibodies against the N1 of the 2009 pandemic H1N1 strain was performed to test the induction of antibodies against neuraminidase (NA). The NA content of influenza virus vaccines is not standardized and usually not measured, even though anti-NA antibodies have been shown to contribute to protection from infection (24, 25). A potent immune response against NA is generally not expected with conventional vaccines (26). The H5N1 vaccine strain contains an NA of the same subtype as H1N1pdm09, and the two proteins share 86.9% amino acid sequence identity. Despite the differences, the adjuvanted H5N1 group (2.2-fold antibody induction; 95% CI, 1.2 to 4.2) and nonadjuvanted H5N1 group (1.4-fold GMI; 95% CI, 1.1 to 1.6) showed low-level antibody induction similar to that in the TIV recipients (1.5-fold GMI; 95% CI, 1.2 to 1.8) (Fig. 1D).

### Serum samples from adjuvanted H5N1 vaccinees protect mice from weight loss after lethal H1N1pdm09 challenge.

**FIG 2:**
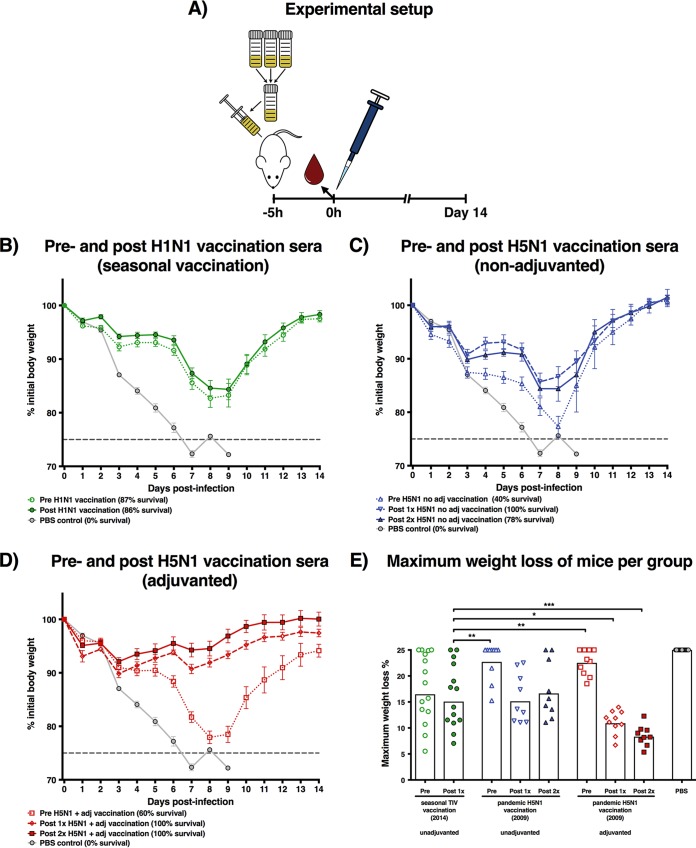
Serum transfer and H1N1 mouse challenge setup and results. (A) Serum samples for each group and time point were pooled, and 150 µl was intraperitoneally injected into mice. Five hours later, animals were sedated, retroorbitally bled to confirm successful serum transfer, and intranasally infected with H1N1pdm09 virus. A negative-control group that received PBS was included and is shown in all weight loss graphs. Mice were monitored daily for weight loss for 14 days. Points indicate group means, and error bars show the standard errors of the means. (B) Weight loss curves for mice that received pooled sera from pre- or post-seasonal influenza vaccination are shown. Percent survival is indicated for each group in the figure legend. (C) Weight loss curves for mice that received pooled sera from prevaccinated, post-1×, or post-2× nonadjuvanted H5N1 vaccination are shown in blue. Percent survival is indicated for each group in the figure legend. (D) Weight loss curves for mice that received pooled sera from prevaccination or post-1× or post-2× adjuvanted H5N1 vaccination are shown. Percent survival is indicated for each group in the figure legend. (E) The maximum weight loss throughout the 14-day observation period was calculated for all animals. Mice that died were assigned the maximum weight loss percentage (25%). Each dot represents a single animal, and white bars show the group geometric mean titers. All groups were compared in a one-way ANOVA Dunnett’s multiple-comparisons test for the post-seasonal influenza vaccination group (standard of care). *, *P* ≤ 0.05; **, *P* ≤ 0.01; ***, *P* ≤ 0.001.

Serum pools were generated for each vaccination group and for different time points. The sera were then intraperitoneally transferred into naive mice. Five hours after the transfer, serum samples were obtained to confirm the presence of human IgG, and the mice were challenged with a lethal dose of H1N1pdm09 (10^3^ PFU) (Fig. 2A). The challenge dose was selected after a dose-finding study indicated that it would successfully induce weight loss (but limited mortality) in mice that received sera from H1N1-primed individuals (Fig. S2). This setup made it possible to detect differences in protection between groups with higher or lower antibody titers.

10.1128/mBio.01463-17.2 FIG S2  H1N1pdm09 challenge dose finding. To establish a challenge dose that would allow detection of differences in weight loss between groups of mice that received human sera with different levels of influenza virus-specific antibodies, a dose-finding experiment was performed. BALB/c mice received either 150 µl of PBS (A) or 150 µl of pooled sera from 30 18- to 20-year-old, deidentified human donors (B), followed by challenge with escalating doses of H1N1pdm09. Points indicate the mean of each group (*n* = 3/group), and error bars show the standard errors of the means. Download FIG S2, TIF file, 0.5 MB. Copyright © 2017 Jacobsen et al. 2017 Jacobsen et al. This content is distributed under the terms of the Creative Commons Attribution 4.0 International license.

Weight loss was observed in both the pre- and post-TIV vaccination serum groups, but the majority (>85%) of mice survived the challenge. Mice that received pre- or post-TIV vaccination serum exhibited similar levels of weight loss (Fig. 2B).

Mice that received pre-H5N1 vaccination serum had lower rates of survival than the pre-TIV vaccination serum group (Fig. 2C and D). Serum after nonadjuvanted H5N1 vaccination conferred increased protection from morbidity after H1N1pdm09 infection compared to baseline serum (Fig. 2C). Interestingly, the groups of mice that received serum after adjuvanted H5N1 vaccination showed 100% survival and good protection from morbidity compared to those that received prevaccination sera (Fig. 2D).

To compare the protective effects of the sera from different vaccination regimens, the maximum weight loss was calculated for each mouse. Dead animals were assigned a value of 25%, the maximum humane weight loss approved by the Institutional Animal Care and Use Committee (IACUC) protocol. All groups were compared to the post-TIV vaccination (standard of care) serum group in a one-way analysis of variance (ANOVA) (Fig. 2E). Consistent with low antibody titers measured by ELISA, both the nonadjuvanted and adjuvanted pre-H5N1 vaccination groups showed significantly higher maximum weight loss than found in the standard of care group (*P* = 0.0024 and *P* = 0.0045, respectively). Mice that received human serum after either one or two adjuvanted H5N1 vaccinations showed significantly less weight loss than the standard of care group (*P* = 0.0414 and *P* = 0.0007, respectively).

### Antibodies induced by seasonal vaccination confer more neutralizing, but less antibody-dependent, cellular cytotoxic activity than antibodies induced by adjuvanted H5N1 vaccination.

**FIG 3:**
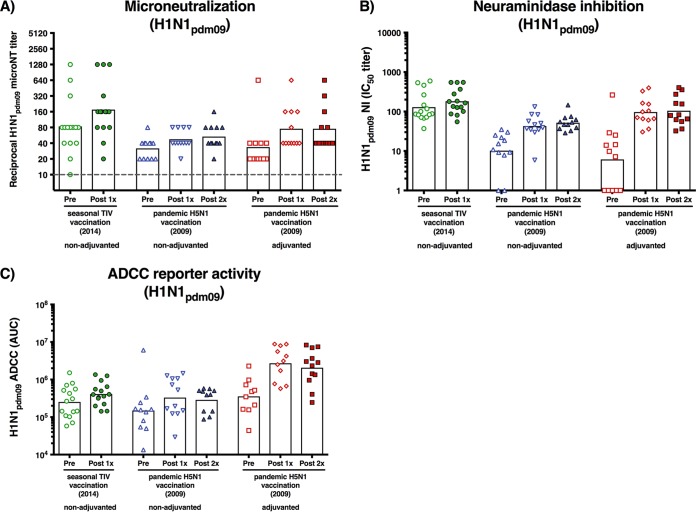
Microneutralization, neuraminidase inhibition, and ADCC reporter assay results. Serum samples from individuals pre- and postvaccination were measured individually. Results for serum samples are shown as individual symbols. Bars represent geometric mean titers. (A) Microneutralization was measured against H1N1pdm09 virus. The gray dashed line indicates the limit of detection. (B) Neuraminidase inhibition was measured against H1N1pdm09 virus in an ELLA. (C) ADCC was measured in an FcγRIIIa-expressing Jurkat cell line-based luciferase reporter assay.

To elucidate the mechanism through which the antibodies conferred protection, we tested the serum samples in additional functional *in vitro* assays. First, the samples were tested in a traditional microneutralization assay against H1N1pdm09. Samples from the seasonal vaccination group had a GMT of 83.8 (95% CI, 42.1 to 166.7) that increased 2.1-fold (95% CI, 1.4 to 3.1) to a GMT of 175.5 (95% CI, 89.1 to 345.9) postvaccination. The H5N1 vaccination groups showed similar induction but had lower pre- and postvaccination GMTs than the TIV group (Fig. 3A). Antibodies against the HA head have previously been shown to potently neutralize viruses, which could explain the higher titers in the TIV group (27). Of note, one individual in the adjuvanted H5N1 vaccination arm who had HI titers of 1:80 at all three time points also showed the highest neutralization titers in that group (1:620). Neutralization can contribute to *in vivo* protection, but the results of the *in vivo* experiment (showing high protection conferred by post-adjuvanted H5N1 vaccination sera) indicated that additional mechanisms are likely to be involved.

Next, we tested the effect of the sera on neuraminidase inhibition in an enzyme-linked lectin assay (ELLA). This assay measures functional inhibition of the neuraminidase activity of a virus by using antibodies. The patterns between the groups were similar to the microneutralization results (Fig. 3B). TIV vaccination resulted in a 1.4-fold increase (95% CI, 1.2 to 1.6) to a geometric mean 50% inhibitory concentration (IC_50_) titer of 181.8 (95% CI, 135.8 to 335.2). Both nonadjvuanted and adjuvanted H5N1 vaccination potently increased NA-inhibiting antibodies (5.1-fold and 17.1-fold, respectively), but the geometric mean IC_50_ titers were lower than those of the standard of care treatment group (52.1 and 104.6, respectively).

Finally, we tested the serum samples in an antibody-dependent cellular cytotoxicity (ADCC) reporter assay. This assay measures the ability of antigen-bound antibodies to engage the human Fc-receptor FcγRIIIa, which can serve as a surrogate for the ability of antibodies to mediate Fc receptor-mediated protection *in vivo*. The results of the ADCC reporter assay were distinctly different from the results of the other functional assays (Fig. 3C). While there was a 1.6-fold GMI (95% CI, 1.1 to 2.3) in the TIV group postvaccination, the adjuvanted H5N1 vaccination group showed significantly higher levels of induced ADCC activity than the TIV group, after either one (*P* < 0.0001) or two vaccinations (*P* = 0.0002). Importantly, these were the groups that showed more *in vivo* protection than the TIV group in the serum transfer experiment (Fig. 2E).

### HA-specific antibodies measured by ELISA correlate with protection *in vivo.*

**FIG 4:**
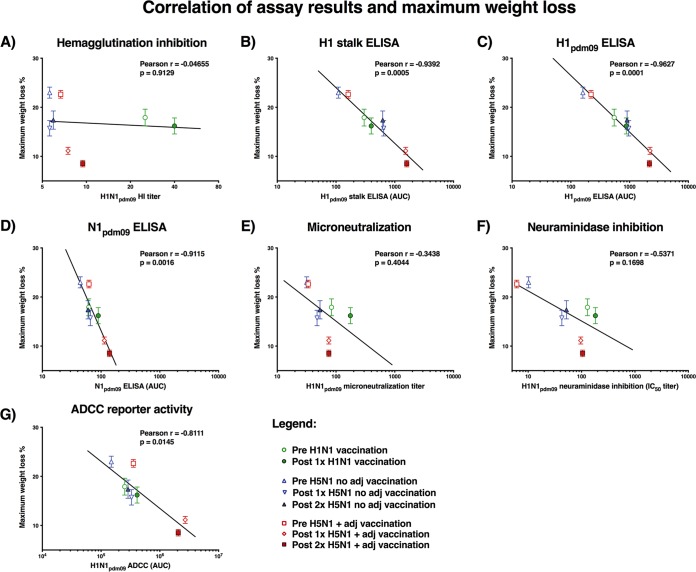
Correlation of assay results and maximum weight loss. The mean maximum weight loss and standard error of the mean are plotted on the *y* axis. The geometric mean titers are plotted on the *x* axis for each group. Each symbol represents one time point of one group. Semilog lines (*y* axis is linear, *x* axis is logarithmic) were plotted for each graph and are shown as black solid lines. Pearson *r* and *P* values for the correlation analysis are shown for each graph. To adjust for multiple comparisons, the *P* values were analyzed with the two-stage step-up method of Benjamini, Krieger, and Yekutieli with a false-discovery rate of 5%. H1 stalk ELISA (*q* = 0.0008), H1pdm09 ELISA (*q* = 0.0003), N1pdm09 ELISA (*q* = 0.0017), and ADCC (*q* = 0.0114) were confirmed as discoveries.

Next, we correlated the *in vitro* assay results with protection *in vivo* (measured as the percent maximum weight loss). HAI antibodies against H1N1pdm09 did not correlate with protection *in vivo* (Fig. 4A). This finding was consistent with the lack of HAI-active antibodies in 6 of the 8 groups that were tested. The antibody levels against both the HA stalk and the full-length H1, measured via an ELISA, correlated well with protection (Fig. 4B and C). Interestingly, antibody levels against the NA also showed a correlation with *in vivo* protection, but the dynamic range was very small (Fig. 4D); ranging from only 44.2 to 138.8 [geometric mean]). In comparison, the antibody levels measured for H1 in an ELISA showed a range of more than 100-fold from lowest to highest titer. A small dynamic range can decrease the predictive value of a result due to the impact of potential assay variation. Both the microneutralization and NA inhibition results showed a general trend with more protection conferred by higher titers (Fig. 4E and F). However, both assays underestimated the protective effect of the sera post-adjuvanted H5N1 vaccination, and neither assay showed a significant correlation. Interestingly, the results of the ADCC reporter assay did correlate with protection *in vivo* (Pearson *r* = −0.8111, *P* = 0.0145), albeit not as well as the results measured by ELISA (Fig. 4G). To test if the significant results were real discoveries or were artifacts due to multiple comparisons, the *P* values were tested with the two-stage step-up method of Benjamini, Krieger, and Yekutieli with a false-discovery rate of 5% (28). The analysis confirmed the results of all three ELISAs as well as the ADCC assay.

## DISCUSSION

Recent advances in influenza virus vaccine research have enabled the development of novel vaccination strategies that target highly conserved epitopes in the influenza hemagglutinin stalk domain. Importantly, antibodies elicited by these vaccines are not detectable by the traditional HAI assay, which is commonly used to measure the efficacy of vaccines. A number of studies have shown that additional assays that measure immune responses to influenza viruses could improve the prediction of protection, including measuring neuraminidase inhibition or cellular responses (9, 24, 25, 29).

In this study, we measured the antibody responses after vaccination with two different influenza virus vaccines. Groups of 15 individuals received either an adjuvanted or a nonadjuvanted avian H5N1 vaccine. We had previously shown that vaccination with vaccines based on avian subtypes that contain an exotic HA head domain and an HA stalk domain that is similar to that of circulating influenza viruses can potently induce stalk-reactive antibodies in humans (21). These vaccination strategies can serve as a surrogate for novel vaccine approaches that elicit HA stalk-based immunity. These novel vaccine approaches aim at shifting antibody responses away from the immunodominant HA head and toward conserved epitopes in the HA stalk (12–14). Importantly, the trial was performed before the novel 2009 H1N1 pandemic virus emerged in Norway, and the participants likely did not have preexisting HAI-reactive antibodies (19, 20). A group of health care workers who received a seasonal trivalent influenza virus vaccine in 2014 was included as a standard-of-care control.

As expected, the traditional HAI assay did not measure an increase in antibody responses against H1N1pdm09 in H5N1-vaccinated individuals. However, an ELISA against the homologous H1 showed potent induction of antibodies, which were confirmed to mainly bind the HA-stalk domain. Post-H5N1 vaccination sera also showed increased activity in neutralization and neuraminidase inhibition assays. Most importantly, we observed a substantial increase in activity in an ADCC reporter assay, which was the only functional assay that reflected the results measured by ELISA. ADCC activity measured in sera from adjuvanted H5N1-vaccinated individuals was higher than in individuals who received the seasonal vaccine with the matched H1N1pdm09 strain. This was consistent with previously published data showing that HA stalk-reactive antibodies potently induce ADCC while HA head-reactive antibodies can interfere with effector cell activation. In addition, antibodies against other influenza virus epitopes likely contribute to the observed ADCC activity (30, 31).

Another important observation was the lack of further induction of HA stalk-specific antibodies and ADCC activity after a second H5N1 vaccination. This is consistent with previous studies in which it was shown that a second vaccination with the same HA head domain shifted the plasmablast response back to its immunodominant epitopes (22). This can be circumvented by vaccination strategies that repeatedly change the HA head domains or by vaccination with headless HA constructs (12–14).

When we tested the *in vivo* protective effect of pooled sera from the study, we found that sera of adjuvanted H5N1 virus vaccinees were highly protective against challenge with H1N1pdm09 in the mouse model, which confirmed that high levels of HA stalk-reactive antibodies could be protective in the absence of HAI-reactive antibodies. When we correlated the maximum weight loss for each group with the GMT measured in binding and functional assays, we found a strong negative correlation with HA-specific antibodies measured via ELISA (Pearson *r* = −0.9627) and a good negative correlation with the ADCC reporter assay results (Pearson *r* = −0.8111), but no correlation with the traditional HAI assay was detected. Importantly, it was previously shown that human IgGs can interact with murine Fc receptors and can confer protection through Fc-mediated mechanisms (32, 33). While the protective effect of human IgG in mice might be not only mediated by ADCC but also by other Fc-mediated mechanisms, the observed correlation indicates that the ADCC reporter assay is a useful surrogate for Fc-mediated protection *in vivo*.

This study has several limitations that need to be considered. The serum volumes available from the clinical trial were finite, which made it necessary to use serum pools to assess *in vivo* protection in mice. Individual differences between subjects could therefore not be assessed. In addition, the contribution of cell-mediated immunity was not tested in our experimental setup. Furthermore, the individuals in the seasonal vaccination group had generally low levels of HAI-reactive antibodies. Therefore, the study cannot properly assess if ELISAs accurately predict the protective effect of high titers of HA head-specific antibodies.

To further develop new correlates of protection, it will be important to expand human challenge trials and test current and novel vaccination approaches while also collecting relevant information on antibody-mediated, cellular, and mucosal immunity. In addition, specimens that were collected during previous epidemiological trials could be tested in the novel assays.

In conclusion, we found that the traditional HAI assay did not accurately predict the protective effect of polyclonal sera in a serum transfer challenge model, when the majority of the antibody response was directed toward conserved epitopes in the HA stalk. Antibodies against these conserved epitopes can be measured by ELISA, and we showed that these antibody levels correlated with protection in the *in vivo* mouse model. These results support further research efforts to develop ELISA-based methods for measuring the immunogenicity and effectiveness of novel universal influenza virus vaccines. Importantly, epidemiological studies should be performed to establish the HA-stalk ELISA titer which affords protection.

## MATERIALS AND METHODS

### Cells, viruses, and proteins.

Dulbecco’s modified Eagle’s medium (DMEM; Life Technologies, Inc., Thermo Fisher Scientific) and UltraMDCK (Lonza) serum-free medium were used to culture Madin-Darbin canine kidney (MDCK) cells. DMEM was supplemented with 10% fetal bovine serum (FBS; Sigma-Aldrich) and an antibiotics mixture (penicillin [100 U/ml] and streptomycin [100 U/ml]; Pen-Strep; Gibco). UltraMDCK medium was supplemented with Pen-Strep (10 U/ml).

Serum-free SFX medium (HyClone) with Pen-Strep (100 U/ml) was used to grow BTI-TN-5B1-4 (High Five) cells (Vienna Institute of Biotechnology subclone) (34). A/Netherlands/602/2009 (H1N1pdm09) virus was grown in 8- to 10-day-old embryonated chicken eggs (Charles River Laboratories, Inc.) at 37°C for 48 h. Infected eggs were chilled to 4°C for 3 h, harvested, and sterile clarified by 0.22-µm filtration (EMD Millipore). The H1N1pdm09 titer was then determined by plaque assay on MDCK cells in the presence of tosyl phenylalanyl chloromethyl ketone (TPCK)-treated trypsin.

Recombinant hemagglutinin and neuraminidase from A/California/04/2009 (Cal09; H1N1pdm09) virus and the chimeric 6/1 hemagglutinin (cH6/1; H6 head domain from A/mallard/Sweden/81/02 combined with an H1 stalk domain of A/California/04/2009) were expressed in BTI-TN-5B1-4 cells and then purified as previously described (26, 35, 36). Briefly, BTI-TN-5B1-4 cells were infected with recombinant baculoviruses expressing soluble proteins at a multiplicity of infection (MOI) of 10. The expressed recombinant proteins were then purified using nickel-nitrilotriacetic acid (Ni-NTA) resin (Qiagen) as previously described (36).

### Human serum samples.

Serum samples from two vaccination trials were used in this study. A cohort of Norwegian health care workers (*n* = 15; 26 to 58 years old, mean age 37.3 years; 20% males) received licensed trivalent inactivated seasonal influenza virus vaccine (15 µg of HA per strain) without adjuvant. The H1N1 component contained the surface glycoproteins of an H1N1pdm09-like virus. The trial was performed in 2014 at the Haukeland University Hospital in Bergen, Norway (NCT01003288), with serum samples being collected prevaccination (day 0) and postvaccination (day 21).

The participants in the H5N1 vaccination trial (*n* = 24; 20 to 48 years, mean age 31.9 years; 33.3% males) received inactivated influenza virus vaccine strain RG14 (surface glycoproteins derived from A/Vietnam/1194/2004 [H5N1] with internal genes from A/Puerto Rico/8/1934 [H1N1]). The vaccine was given in two doses with a 21-day interval between the first and second dose (30 µg HA/dose). Half of the participants received the vaccine adjuvanted with Matrix M (AbISCO-100; Isconova AB) (37). The trial was performed in early 2009 at the Haukeland University Hospital in Bergen, Norway, with samples being collected prevaccination (day 0), postvaccination after one dose (day 21), and postvaccination after the second dose (day 42). Additional information for the clinical trial can be found on ClinicalTrials.gov website (NCT00868218) identifier (20, 21, 38).

### Receptor-destroying enzyme treatment of sera.

Human serum samples were treated with receptor-destroying enzyme (RDE; Denka Seiken) according to the manufacturer’s instructions, with an additional inactivation step. Briefly, one part serum sample was mixed with three parts RDE solution (reconstituted with phosphate-buffered saline [PBS]) and incubated at 37°C for 18 h. RDE was inactivated with 3 volumes (based on original serum volume) of 2.5% sodium citrate solution and then incubated at 56°C for 30 min. Additionally, three volumes of PBS based on original serum volume were added for a final human serum sample concentration of 1:10.

### Hemagglutination inhibition assay.

The HAI assay was performed as previously described (39). Briefly, RDE-treated human serum samples were serially diluted 1:2 in V-bottom 96-well microtiter plates (Thermo Fisher Scientific), with a starting concentration of 1:10. Twenty-five-microliter aliquots of diluted serum samples were then incubated with 4 HA units of virus in 25 µl of PBS. After 30 min of incubation at room temperature, 50 µl of 0.5% chicken red blood cells (Lampire Biological Laboratories) in PBS were added to the virus-serum mixture. The HAI activity was read after a 45-min incubation at 4°C. A value of 5 was assigned to samples below the limit of detection for the statistical analysis.

### ELISA.

Flat-bottom 96-well plates (Immulon 4 HBX; Thermo Fisher Scientific) were coated with 2 µg/ml protein (50 µl/well) diluted in coating solution (KPL) overnight at 4°C. The following day, plates were washed three times with PBS containing 0.1% Tween 20 (Fisher Scientific) (T-PBS) and were blocked with 220 µl of blocking solution (3% goat serum [Gibco] and 0.5% milk powder in T-PBS) for 1 h at room temperature. After removal of the blocking buffer, serum samples at a 1:100 starting concentration were 2-fold serially diluted in blocking solution. After 2 h of incubation at room temperature, the plates were washed three times with T-PBS. Anti-human IgG (Fab specific) horseradish peroxidase (HRP)-conjugated antibody (Sigma-Aldrich) diluted 1:3,000 in blocking solution was added to all wells (50 µl/well). After a 1-h incubation at room temperature, the plates were washed four times with T-PBS and developed with 100 µl/well of SigmaFast *ο*-phenylenediamine dihydrochloride (OPD; Sigma) for 10 min. The reaction was stopped with 50 µl of 3 M hydrochloric acid (HCl) and then subsequently read at an absorbance of 490 nm with a Synergy H1 hybrid multimode microplate reader (BioTek). The average background plus three standard deviations were calculated and used as the lower limit for area under the curve (AUC) analysis using GraphPad Prism software.

### Microneutralization assay.

Flat-bottom 96-well cell tissue culture plates (Corning Costar) were seeded in 100 µl of UltraMDCK serum-free culture medium containing 2.0 × 10^5^ MDCK cells and incubated at 37°C overnight. The following day, RDE-treated serum samples diluted to 1:10 were 2-fold serially diluted in UltraMDCK containing TPCK-treated trypsin (infection medium) in a separate 96-well tissue culture plate. Diluted sera were incubated with H1N1pdm09 virus (200 PFU/50 µl) for 1 h at room temperature with shaking. Cell-containing 96-well plates were washed with PBS, and 100 µl of the virus-sera mixture was added. After a 1-h incubation at 37°C the mixture was removed, cells were washed with PBS, and 50 µl of serially diluted serum plus 50 µl infection medium were added to each well. The cells were then incubated at 37°C for 72 h. To measure microneutralization activity, 50 µl of the supernatant was transferred into a separate V-bottom 96-well plate, and 50 µl of 0.5% chicken red blood cells in PBS were added. The samples were read after 45 min of incubation at 4°C. A value of 5 was assigned to samples below the limit of detection for statistical analysis.

### Enzyme-linked lectin assay (ELLA).

The ELLA was performed according to previously published protocols with slight modifications (40, 41). First, a neuraminidase assay was performed to identify the optimal virus concentration to be used in the ELLA. Microtiter 96-well plates were coated with 100 µl fetuin (Sigma-Aldrich) at 50 µg/ml diluted in coating solution (KPL) and incubated at 4°C overnight. The following day, viral stocks were serially diluted 2-fold in PBS containing 5% bovine serum albumin (BSA; blocking buffer) in a separate 96-well plate, starting undiluted, and incubated at room temperature for 90 min. The fetuin-coated plates were washed three times with T-PBS and blocked with 200 µl blocking buffer for 1 h at room temperature. Subsequently, the blocked plates were washed three times with T-PBS, and 100-µl aliquots from the plates containing virus were transferred to the fetuin-coated plates. The plates containing virus and fetuin plates were incubated at 37°C for 2 h and then washed three times with T-PBS. Peanut agglutinin (Po-PNA) conjugated to HRP (Sigma-Aldrich) at 5 µg/ml (100 µl/well) diluted in PBS was added to all wells, and the plates were incubated for 90 min at room temperature in the dark. The plates were washed four times, and 100 µl of OPD (Sigma-Aldrich) was added to all wells for development. The reaction was stopped after 5 min by adding 50 µl of 3 M HCl to all wells. The plates were read at an absorbance of 490 nm with a Synergy H1 hybrid multimode microplate reader (BioTek). To determine the optimal virus concentration to be used in the ELLA, the absorbance data were plotted using GraphPad Prism. A nonlinear regression curve was then fit to determine the 50% effective concentration (EC_50_). Two times the EC_50_ (2× EC_50_) was used in subsequent NI assays.

For ELLAs, microtiter 96-well plates (Immulon 4 HBX; Thermo Fisher Scientific) were coated as described above and incubated overnight at 4°C. The following day, RDE-treated human serum samples (with a starting concentration of 1:10) were serially diluted 1:2 in PBS in a separate 96-well plate, resulting in a final volume of 75 µl/well. Virus stocks were then diluted in PBS to the determined 2× EC_50_ level. Diluted virus (75 µl/well) was then added to the serum plates and incubated at room temperature for 90 min. In the meantime, fetuin-coated plates were washed and blocked as described above. After blocking, the fetuin-coated plates were washed three times with T-PBS, 100 µl of serum-virus mixture was added, and the mixture was incubated for 2 h at 37°C. The plates were washed four times with T-PBS, and Po-PNA conjugated to HRP (100 µl/well at 5 µg/ml diluted in PBS) was added. All additional steps were performed as described above. To determine human serum reactivity, background absorbance values were subtracted from the raw absorbance values of the human serum samples. The difference was then divided by the average value of the virus-only control well and then multiplied by a factor of 100 to calculate the percent neuraminidase activity. Percent neuraminidase inhibition in the presence of human serum samples was determined by subtracting neuraminidase activity in each well from 100%. The percent neuraminidase inhibition was then fit to a nonlinear regression curve using GraphPad Prism to determine the IC_50_ titers of the human serum samples (40, 41).

### Antibody-dependent cellular cytotoxicity reporter assay.

The ADCC reporter assay was performed as previously described (16, 42). In brief, 96-well white flat-bottom plates (Costar Corning) were seeded with 100 µl of complete Dulbecco’s modified Eagle medium containing 2 × 10^5^ MDCK cells. After 24 h of incubation at 37°C, the MDCK cells were washed once with PBS and then infected with H1N1pdm09 virus at an MOI of 1. The infected cells were incubated at 33°C for 24 h. The next day, in a separate 96-well plate, non-RDE-treated human serum samples were serially diluted 2-fold in RPMI 1640 medium (Gibco, Thermo Fisher Scientific) with a starting concentration of 1:50. ADCC bioassay effector cells (Jurkat V variant cells; Promega) were diluted to 3 × 10^6^ cells/ml in RPMI 1640 medium. The supernatant from the infected cell plate was aspirated, and fresh RPMI medium (25 µl/well) was added. Furthermore, diluted serum samples (25 µl/well) from the 96-well dilution plate and diluted ADCC bioassay effector cells (25 µl/well) were also added to the infection plate. After incubation at 37°C for 6 h, Bio-Glo luciferase assay reagent (75 µl/well; Promega) was added, and luminescence was measured using a Synergy H1 hybrid multimode microplate reader (BioTek). The average background plus five standard deviations was calculated and used as the lower limit for AUC analysis using GraphPad Prism. One baseline and one day 42 sample were not available for testing in the nonadjuvanted H5N1 vaccination group. Two baseline and one day 21 sample were not available for testing in the adjuvanted H5N1 vaccination group.

### Serum transfer study and virus challenge.

To establish a challenge dose of H1N1pdm09 for mice that received sera from human adults, a dose-finding experiment was performed. Fifteen mice were intraperitoneally injected with 150 µl of PBS, and 15 mice received 150 µl of pooled serum collected in 2014 from 30 18- to 20-year-old, deidentified healthy human donors (Innovative Research). The mice were then sedated, split into groups of 3 animals, and challenged with escalating doses of H1N1pdm09 virus (10 to 10^5^ PFU) in 50 µl of PBS.

To assess the protective effect of sera from vaccinated individuals against H1N1pdm09 virus challenge in mice, transfer of pre- and postvaccination human sera followed by a virus challenge was performed. Human sera were pooled for each vaccination trial and for different time points. The serum pools were generated by mixing equal serum volumes from each individual of a cohort for each time point. The size of a pools was limited by the lowest individual sample volume available for each cohort. One hundred fifty microliters of serum was injected intraperitoneally into each mouse. Fifteen mice each received pooled sera from individuals pre- and postseasonal vaccination. Serum pools from individuals vaccinated with nonadjuvanted or adjuvanted H5N1 virus were transferred into 10 mice per group and time point. Fifteen mice that received PBS were included as a negative-control group. Five hours after the serum transfer, mice were bled retroorbitally and challenged with 10^3^ PFU of H1N1pdm09 virus. Sera were tested by ELISA for human H1-specific IgG to confirm successful antibody transfer. Mice that did not show positive serum responses were excluded from analysis. Weight loss was observed for 14 days. Mice that reached a body weight less than 75% of their initial weight were euthanized. All mouse experiments were performed in accordance with the guidelines of the Icahn School of Medicine at Mount Sinai Institutional Animal Care and Use Committee (IACUC-2015-0119).

### Statistical analysis.

All statistical analyses were performed using GraphPad Prism. To compare multiple groups in the ADCC reporter assay, an ordinary one-way ANOVA with a Holm-Sidak posttest for multiple comparisons was performed. All groups were compared to the post-1× seasonal vaccination group. Maximum weight loss of mice was compared to that for the group that received post-seasonal influenza vaccination sera in a one-way ANOVA with a Dunnett posttest. Correlations were measured in Pearson correlation tests. To adjust for multiple comparisons, the *P* values for the weight loss correlations were analyzed with the two-stage step-up method of Benjamini, Krieger, and Yekutieli with a false-discovery rate of 5% (28).
