# Supplementary figures and images for: Influenza Virus Hemagglutinin Stalk-Specific Antibodies in Human Serum are a Surrogate Marker for In Vivo Protection in a Serum Transfer Mouse Challenge Model
Source: mBio. 2017 Sep 19;8(5):e01463-17. doi: 10.1128/mBio.01463-17 (PMC5605943; doi:10.1128/mBio.01463-17)

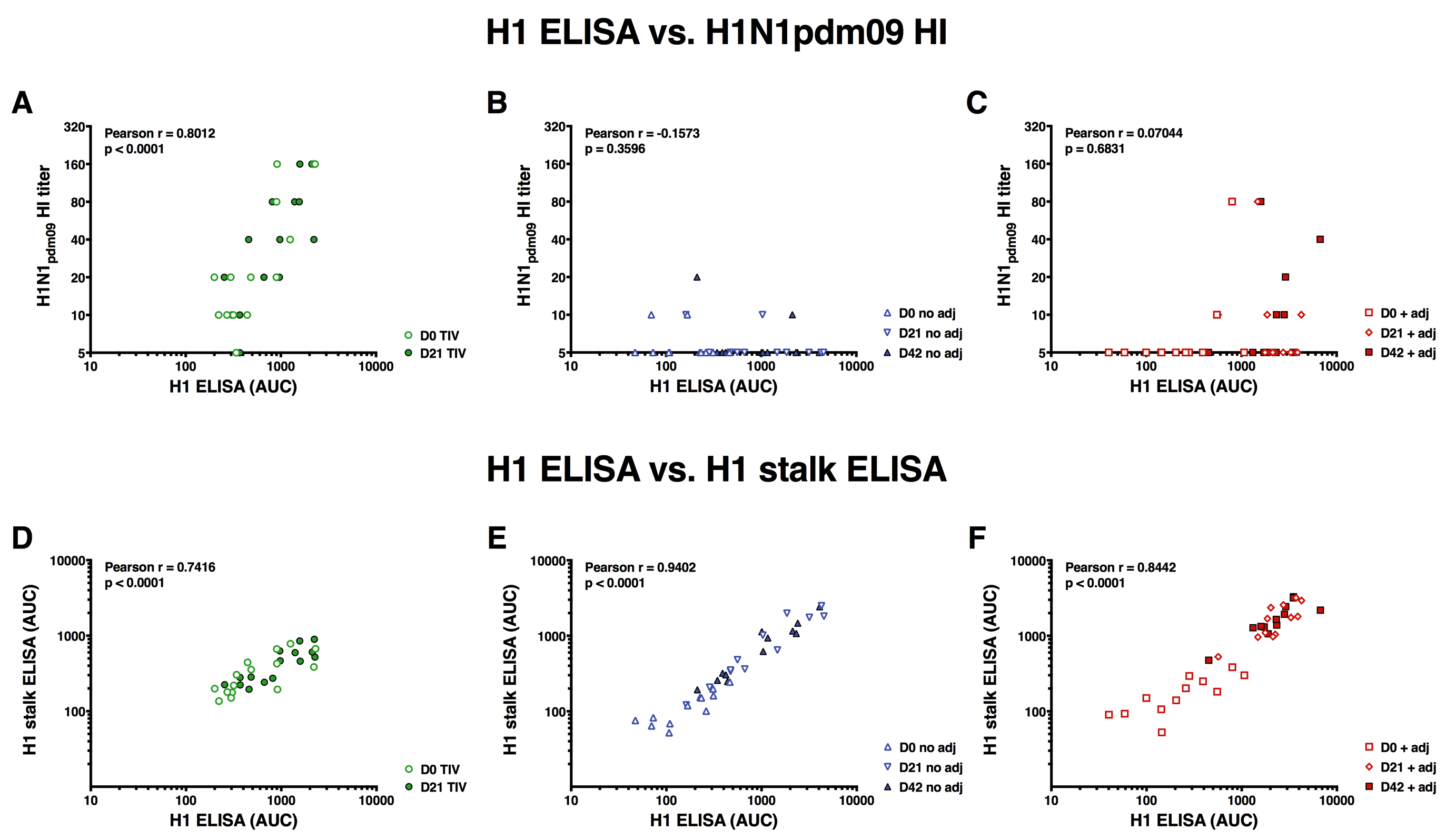

Supplement: FIG S1 [file mbo005173498sf1.tif]

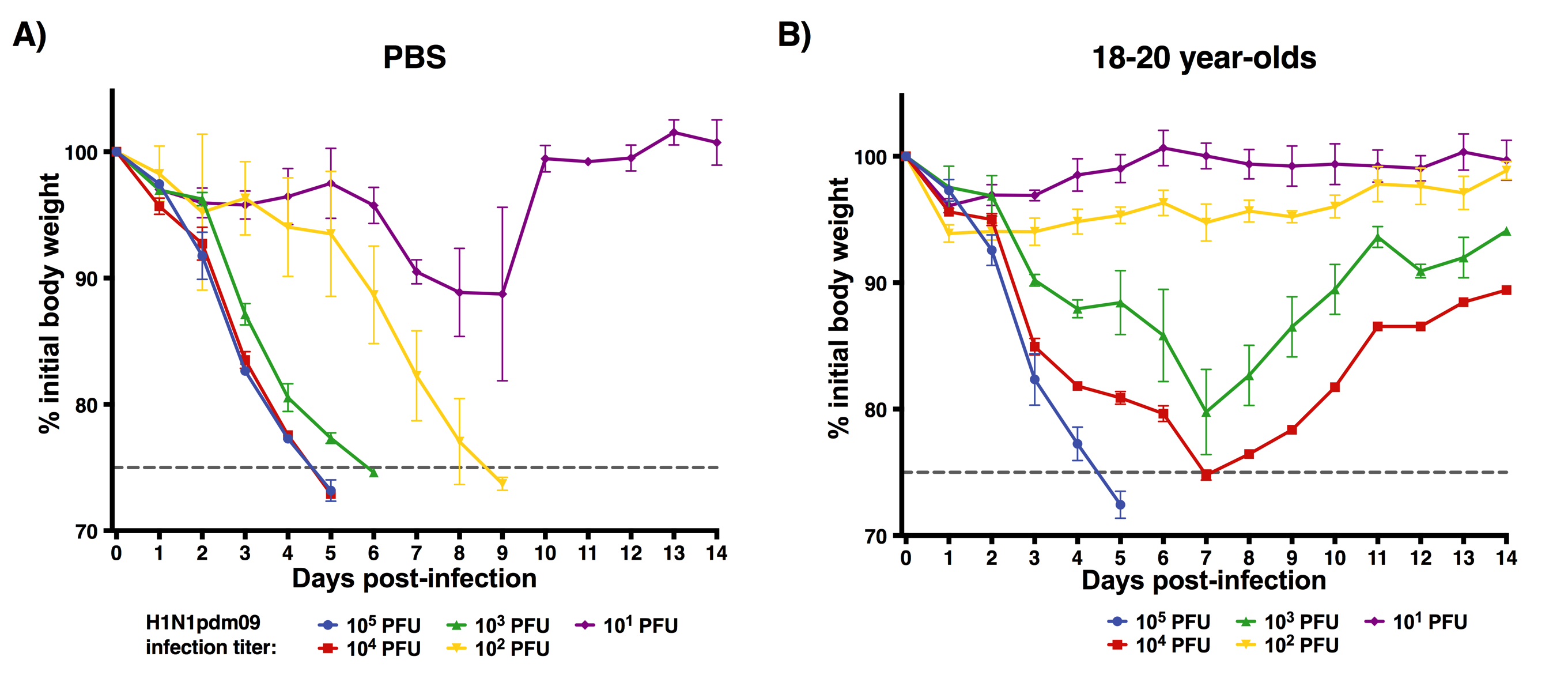

Supplement: FIG S2 [file mbo005173498sf2.tif]
